# Activation of Kinin B1R Upregulates ADAM17 and Results in ACE2 Shedding in Neurons

**DOI:** 10.3390/ijms22010145

**Published:** 2020-12-25

**Authors:** Rohan Umesh Parekh, Srinivas Sriramula

**Affiliations:** Department of Pharmacology and Toxicology, Brody School of Medicine, East Carolina University, Greenville, NC 27834, USA; parekhr13@students.ecu.edu

**Keywords:** ACE2, ADAM17, kinin B1R, glutamate, shedding

## Abstract

Angiotensin converting enzyme 2 (ACE2) is a critical component of the compensatory axis of the renin angiotensin system. Alterations in ACE2 gene and protein expression, and activity mediated by A Disintegrin And Metalloprotease 17 (ADAM17), a member of the “A Disintegrin And Metalloprotease” (ADAM) family are implicated in several cardiovascular and neurodegenerative diseases. We previously reported that activation of kinin B1 receptor (B1R) in the brain increases neuroinflammation, oxidative stress and sympathoexcitation, leading to the development of neurogenic hypertension. We also showed evidence for ADAM17-mediated ACE2 shedding in neurons. However, whether kinin B1 receptor (B1R) activation has any role in altering ADAM17 activity and its effect on ACE2 shedding in neurons is not known. In this study, we tested the hypothesis that activation of B1R upregulates ADAM17 and results in ACE2 shedding in neurons. To test this hypothesis, we stimulated wild-type and B1R gene-deleted mouse neonatal primary hypothalamic neuronal cultures with a B1R-specific agonist and measured the activities of ADAM17 and ACE2 in neurons. B1R stimulation significantly increased ADAM17 activity and decreased ACE2 activity in wild-type neurons, while pretreatment with a B1R-specific antagonist, R715, reversed these changes. Stimulation with specific B1R agonist Lys-Des-Arg^9^-Bradykinin (LDABK) did not show any effect on ADAM17 or ACE2 activities in neurons with B1R gene deletion. These data suggest that B1R activation results in ADAM17-mediated ACE2 shedding in primary hypothalamic neurons. In addition, stimulation with high concentration of glutamate significantly increased B1R gene and protein expression, along with increased ADAM17 and decreased ACE2 activities in wild-type neurons. Pretreatment with B1R-specific antagonist R715 reversed these glutamate-induced effects suggesting that indeed B1R is involved in glutamate-mediated upregulation of ADAM17 activity and ACE2 shedding.

## 1. Introduction

Angiotensin converting enzyme 2 (ACE2) is well established as a critical enzyme of the renin angiotensin system, that can cleave vasoconstrictor peptide angiotensin (Ang)-II into the vasodilatory peptide Ang-(1–7) in many tissues, including heart, kidney, vasculature, and brain [1,2]. ACE2 plays a major role in counterbalancing the vasoconstrictor and proliferative effects of angiotensin (Ang) II by reducing Ang II-mediated responses and increasing the vasodilatory and anti-proliferative effects of Ang-(1–7) [1]. The ACE2/Ang-(1–7) pathway plays an important role in opposing the effects of Ang II and its major receptor Ang II type 1 receptor (AT1R) on pathological processes such as inflammation, oxidative stress, and neurodegeneration [1,3]. Alterations in ACE2 gene and protein expression, and activity are implicated in several cardiovascular and neurodegenerative diseases. There is significant evidence that decreased levels of ACE2 are associated with conditions such as hypertension in human patients and animal models. On the other hand, potential therapeutic benefits of increasing ACE2 expression or activity using recombinant human ACE2, gene therapy, or ACE2 activators has been shown to be effective in several diseases including hypertension, heart failure, stroke, diabetes, and kidney injury [1,4,5,6]. ACE2 is a cell membrane-bound enzyme with its catalytic site exposed to the extra cellular surface [7]. ACE2 levels within tissues and cells can be modified by post translational mechanisms such as ACE2 ectodomain shedding and AT1R-dependent internalization processes [5,6,8,9]. A prime mediator of ACE2 shedding is A Disintegrin And Metalloprotease 17 (ADAM17), a member of the “A Disintegrin And Metalloprotease” (ADAM) family, also known as tumor necrosis factor-alpha converting enzyme [10,11,12]. ADAM17 is an ACE2 sheddase that is upregulated following activation of AT1R and is responsible for the impairment of ACE2 compensatory function in the mouse hypothalamus during neurogenic hypertension [5]. In addition, we have previously demonstrated that ACE2 ectodomain shedding takes place in human brains, and ADAM17-mediated ACE2 shedding is exacerbated in the brains of hypertensive patients [6]. Recent studies further highlight an important role for ACE2 as a viral receptor in the pathogenesis of severe acute respiratory syndrome corona virus 2 [13,14] and it has been suggested that the delicate balance between ACE2 and ADAM17 interactions could play a critical role in clinical outcome of corona virus disease 19 [15].

Kinins are vasoactive peptides released by the enzymatic action of plasma (KLKB1) and tissue (KLK1) kallikreins on substrates known as kininogens. The family of kinin peptides mediates biological actions by activating two kinin receptors known as kinin B1 receptor (B1R) and kinin B2 receptor (B2R) that belong to the superfamily of G protein-coupled receptors [16,17]. Des-Arg^9^-Bradykinin and Lys-Des-Arg^9^-Bradykinin (LDABK) are the active metabolites of bradykinin and kallidin, respectively, and are the endogenous ligands for the B1R [18]. B1R has a very low expression under native conditions but is highly and rapidly upregulated by inflammatory conditions. Despite its inducible profile, B1R can be found constitutively expressed in the central nervous system [19]. In contrast to B2R, B1R does not undergo internalization following agonist stimulation but tends to translocate and aggregate after agonist binding and facilitate the amplification of B1R-mediated responses. Activation of B1R-mediated signaling pathways is implicated in the pathogenesis of various cardiovascular diseases that are associated with or induced by inflammation [16]. We previously showed that activation of B1R in the brain hypothalamic paraventricular nucleus resulted in elevated neuroinflammation, oxidative stress and sympathoexcitation, leading to the development of neurogenic hypertension [20]. We also demonstrated evidence for ADAM17-mediated ACE2 shedding in the neurons [8]. However, there has been no study investigating the direct interaction between B1R and ADAM17 in inducing ACE2 shedding in neurons.

Glutamate is one of the key excitatory neurotransmitters of the central nervous system. Evidence suggests that the hypothalamus contains high levels of glutamate and plays an important role in cardiovascular and autonomic regulation [21]. Excessive stimulation by glutamate results in excitotoxicity and contributes to the development of inflammatory neurodegeneration through dysregulation of calcium homeostasis and initiating a cascade of signaling pathways, including, dysfunction of mitochondria, reactive oxygen species production, endoplasmic reticulum stress, and NOS mediated oxidative stress mechanisms [22,23]. In addition, glutamate can induce upregulation of several pro-inflammatory cytokines including tumor necrosis factor-alpha [24]. We previously demonstrated that the activity of ADAM17 is elevated during excessive glutamate stimulation, ultimately leading to ACE2 shedding in neurons [8]. However, the crosstalk between glutamate and B1R, and the involvement of B1R activation in ADAM17-mediated ACE2 shedding, have not been investigated.

Therefore, in the present study, we investigated the hypothesis that the activation of B1R will result in ADAM17-mediated ACE2 shedding in primary hypothalamic neurons. In addition, we also determined the crosstalk between excessive glutamate stimulation and ADAM17-mediated ACE2 shedding via upregulation of B1R in neurons.

## 2. Results

### 2.1. B1R Activation Increases ADAM17 Activity in Primary Hypothalamic Neurons

**Figure 1:**
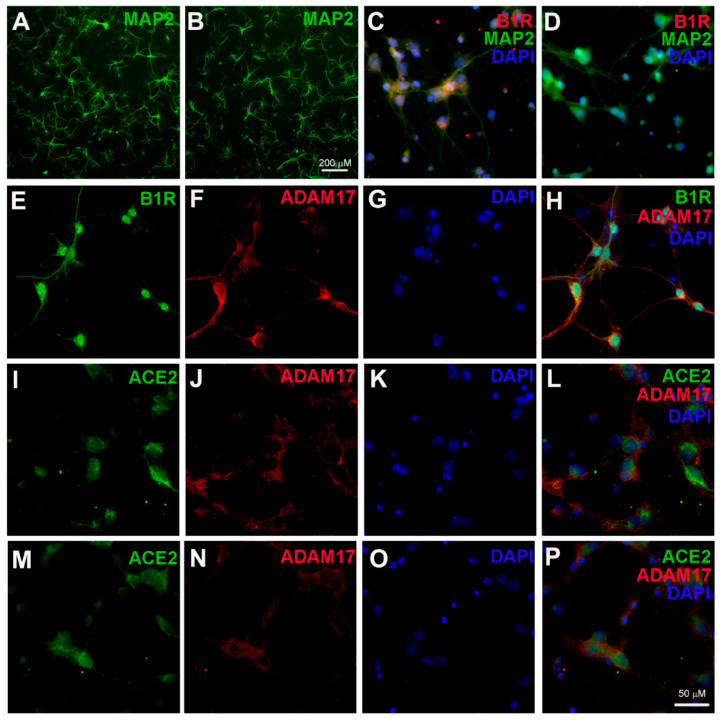
Kinin B1 receptor (B1R), A Disintegrin And Metalloprotease 17 (ADAM17), and angiotensin converting enzyme 2 (ACE2) expression in primary hypothalamic neurons. Representative photomicrographs showing immunofluorescence staining for neuron-specific marker microtubule associated protein 2 (MAP-2) (Green) in primary neurons from wild-type (**A**) and B1R knockout mice (**B**) cultured for 10 days. Primary neurons from wild-type mice pups showed immunopositivity for the presence of B1R (**C**), while B1R immunoreactivity was absent in neurons with B1R deletion (**D**). Representative triple immunostaining revealed that kinin B1R (**E**) and ADAM17 (**F**) are expressed in primary hypothalamic neurons expressing nuclear DAPI staining in blue (**G**), and merged image (**H**) shows the colocalization of B1R and ADAM17. Representative triple immunostaining showing ACE2 and ADAM17 expression and co-localization in wild-type neurons (**I**–**L**) and in B1R knockout neurons (**M**–**P**).

To test the hypothesis that B1R activation can increase ADAM17 expression and activity in neurons, we first confirmed the expression of B1R and ADAM17 in cultured primary hypothalamic neurons. We cultured primary hypothalamic neurons from both wild-type and B1R knockout (B1RKO) mice pups and validated via immunofluorescence labeling with a neuron-specific marker, microtubule-associated protein 2 (MAP2) (Figure 1A,B). The neurons from both wild-type and B1RKO mice pups cultured for 14 days with cytosine arabinofuranoside (Ara-C) treatment showed predominantly neuronal population, demonstrating numerous processes and discrete cellular morphology of neurons with cell–cell interactions. Primary neurons cultured from wild-type mice pups showed immunopositivity for the presence of B1R (Figure 1C), while B1R immunoreactivity was absent in neurons from B1RKO mice pups (Figure 1D). Using triple immunofluorescence staining with validated antibodies, we confirmed the expression of B1R and ADAM17, and their co-localization on cultured wild-type primary neurons (Figure 1E–H). In addition, we also confirmed the expression and co-localization of both ACE2 and ADAM17 in wild-type (Figure 1I–L) and B1RKO (Figure 1M–P) primary hypothalamic neurons.

**Figure 2:**
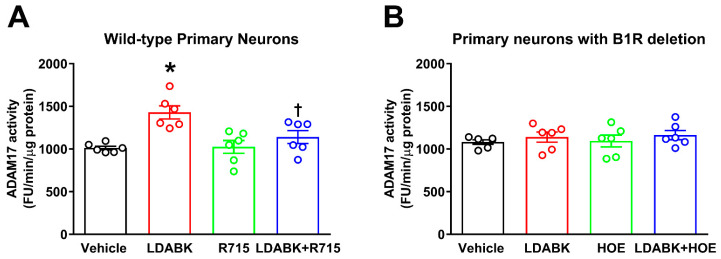
Effect of B1R activation on ADAM17 activity in primary hypothalamic neurons. (**A**) Incubation of wild-type neurons with B1R-specific agonist Lys-Des-Arg^9^-Bradykinin (LDABK) significantly increased ADAM17 activity. Pre-treatment with R715, a B1R-specific antagonist, prevented this LDABK-mediated increase in ADAM17 activity. R715 treatment alone did not have any effect on ADAM17 activity. (**B**) Treatment with LDABK did not show any effect on ADAM17 activity in neurons with B1R deletion. In addition, the HOE 140, a kinin B2 receptor (B2R)-specific antagonist also did not show any effect on ADAM17 activity. The cultured primary neurons were pre-treated with a specific B1R antagonist (R715, 10 μM) or a specific B2R antagonist HOE 140 (HOE, 10 μM) for 1 h, followed by treatment with LDABK (300 nM) for 18 h (*n* = 6 independent cultures/group). Statistical significance: one-way ANOVA followed by Tukey’s multiple comparisons test. * *p* < 0.05 compared to vehicle, † *p* < 0.05 compared to LDABK.

Next, neurons from wild-type and B1RKO mice pups were treated with B1R-specific agonist Lys-des-Arg^9^-BK (LDABK, 300 nM) and ADAM17 activity was measured using a florescent kinetic assay as described previously [8,25]. Incubation of wild-type neurons with B1R-specific agonist LDABK significantly increased ADAM17 activity. Pretreatment with a B1R-specific antagonist, R715 (Ac-Lys-Arg-Pro-Gly-Phe-Ser-DβN al-Ile) prevented this LDABK-mediated increase in ADAM17 activity (Figure 2A). On the other hand, treatment with LDABK did not show any effect on ADAM17 activity in neurons with B1R deletion (Figure 2B). In addition, the HOE 140 (D-Arg-Arg-Pro-Hyp-Gly-Thi-Ser-D-Tic-Oic-Arg), a potent B2R-specific antagonist also did not show any effect on ADAM17 activity. These data clearly suggest that the B1R activation, not B2R, increases ADAM17 activity in neurons.

### 2.2. B1R Activation Results in Reduced ACE2 Activity in Primary Hypothalamic Neurons

**Figure 3:**
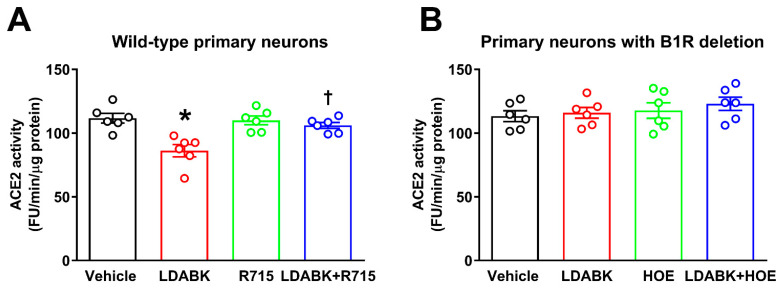
Effect of B1R activation on ACE2 activity in primary hypothalamic neurons. (**A**) Incubation of wild-type neurons with B1R-specific agonist LDABK significantly decreased ACE2 activity. R715, a B1R-specific antagonist pretreatment prevented this LDABK-mediated decrease in ACE2 activity. R715 treatment alone did not have any effect on ACE2 activity. (**B**) Treatment with LDABK did not show any effect on ACE2 activity in neurons with B1R deletion. In addition, the HOE 140, a B2R-specific antagonist also did not show any effect on ACE2 activity. The cultured primary neurons were pre-treated with a specific B1R antagonist (R715, 10 μM) or a specific B2R antagonist HOE 140 (HOE, 10 μM) for 1 h, followed by treatment with LDABK (300 nM) for 18 h (*n* = 6 independent cultures/group). Statistical significance: one-way ANOVA followed by Tukey’s multiple comparisons test. * *p* < 0.05 compared to vehicle, † *p* < 0.05 compared to LDABK.

We previously demonstrated that increased activity of ADAM17 can result in ACE2 ectodomain shedding in the brains of hypertensive mice [5,6] and cultured cortical neurons [8]. However, whether B1R is involved in ACE2 shedding is not known. Therefore, here we tested the hypothesis that B1R activation will mediate increased ADAM17 activity and reduce ACE2 activity. Treatment of wild-type neurons with B1R-specific agonist LDABK for 18 h significantly decreased ACE2 activity (Figure 3A). In addition, pretreatment with R715, a B1R-specific antagonist, prevented this LDABK-mediated decrease in ACE2 activity. We further confirmed our B1R pharmacological antagonist results using neurons with B1R gene deletion. As shown in Figure 3B, stimulation with LDABK did not show any effect on ACE2 activity in neurons with B1R deletion. In addition, HOE 140, a B2R-specific antagonist also did not show any effect on ACE2 activity (Figure 3B). These results indicate that B1R activation results in ADAM17-mediated shedding and reduced ACE2 activity in primary hypothalamic neurons.

### 2.3. Stimulation with Glutamate Induces B1R Expression

**Figure 4:**
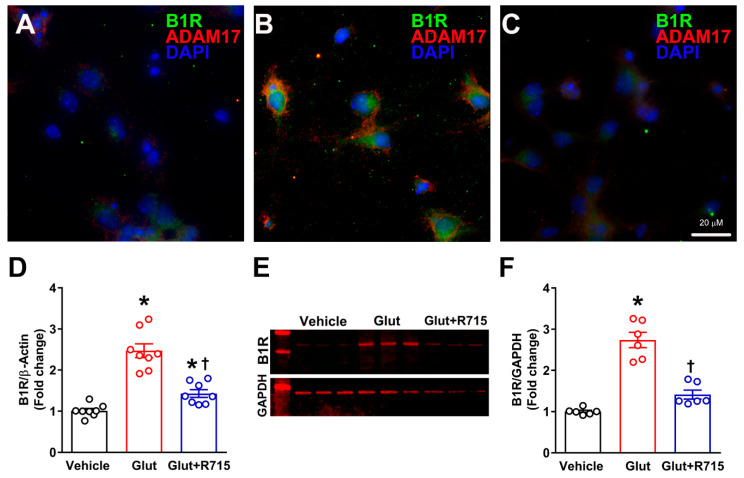
Effect of glutamate on B1R activation in primary hypothalamic neurons. Representative triple immunostaining revealed that compared to vehicle (**A**) treatment, stimulation with glutamate (100 μM) (**B**) for 18 h induced the expression of kinin B1R and ADAM17 protein in primary hypothalamic neurons. This glutamate-induced increase in B1R and ADAM17 protein expression was prevented by pre-treatment with R715 (**C**). Treatment with glutamate (100 μM) for 4 h induced an increase in B1R mRNA in cultured neurons, measured by real time PCR. This increase in B1R mRNA was prevented by pre-treatment with R715 (**D**). Representative image of Western blot (**E**) and quantitative analysis (**F**) showing glutamate stimulation induced B1R protein expression which was prevented by pre-treatment with R715 in neurons (*n* = 4–6 independent cultures/group). Statistical significance: one-way ANOVA followed by Tukey’s multiple comparisons test. * *p* < 0.05 compared vehicle, † *p* < 0.05 compared to glutamate.

To test the hypothesis that stimulation with glutamate results in B1R activation, cultured primary hypothalamic neurons were treated with glutamate (100 μM) for 18 h, and B1R gene and protein expression was measured. We used 100 μM of glutamate concentration for the treatment of neurons, which has been previously shown to increase ADAM17 activity in cortical neurons [8]. Glutamate treatment increased the expression of B1R in neurons along with increased ADAM17 protein compared to vehicle treated neurons as measured by immunofluorescence staining (Figure 4). Furthermore, pretreatment with R715, a B1R-specific antagonist, prevented this glutamate-mediated increase in B1R and ADAM17 expression. As shown in Figure 4D, the B1R gene expression was significantly increased after glutamate treatment for 4 h and this increase was attenuated by pre-treatment with a B1R-specific antagonist. We further confirmed the effect of glutamate on B1R protein expression using Western blot analysis. Representative Western blot (Figure 4E) and quantification of data (Figure 4F) show that similar to gene expression results, glutamate significantly increased the protein expression of B1R in hypothalamic neurons, and this increase was prevented by the treatment with B1R antagonist. These results clearly indicate that glutamate can induce B1R gene and protein expression in neurons.

### 2.4. Glutamate Induces ADAM17-Mediated ACE2 Shedding via B1R Activation 

**Figure 5:**
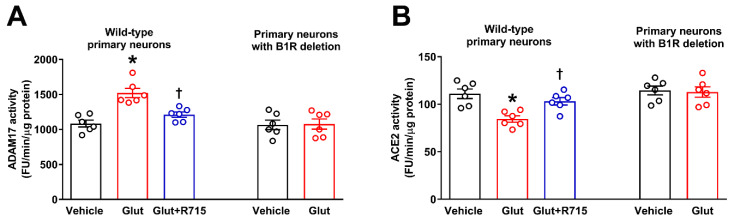
Glutamate-induced ADAM17-mediated ACE2 shedding involves kinin B1R activation. Glutamate increased ADAM17 activity (**A**) and decreased ACE2 activity (**B**) in neurons, which was prevented by pre-treatment with R715. Treatment of B1R knockout neurons with glutamate did not show any effect on ADAM17 or ACE2 activity. ADAM17 and ACE2 assays were performed in primary hypothalamic neurons cultured from wild-type and B1R knockout neonates, treated with glutamate (Glut, 100 μM) or glutamate with 1 h of pre-treatment with R715 (a specific B1R inhibitor, 10 μM) for 18 h (*n* = 6 independent cultures/group). Statistical significance: one-way ANOVA followed by Tukey’s multiple comparisons test. * *p* < 0.05 compared to vehicle, † *p* < 0.05 compared to glutamate.

We previously showed that excessive glutamate (100 μM) stimulation can induce ADAM17-mediated impairment in ACE2 activity in neurons [8]. The involvement of ADAM17 in the glutamate-induced reduction in ACE2 activity was confirmed by pre-treating neurons with DPC333, a specific ADAM17 inhibitor, which fully restored the glutamate-induced reduction in ACE2 activity [8]. To test the hypothesis that B1R activation is involved in glutamate-induced ADAM17-mediated ACE2 shedding, neurons were pre-treated with R715, and then stimulated with glutamate. Glutamate significantly increased the ADAM17 activity (Figure 5A) and reduced the enzymatic activity of ACE2 (Figure 5B) in primary hypothalamic neurons, after 18 h of treatment. In addition, pretreatment with B1R-specific antagonist R715 reversed these glutamate-induced effects on ADAM17 and ACE2 activity. Further, treatment of B1RKO mice neurons with glutamate did not show any increase in ADAM17 activity (Figure 5A) or decrease in ACE2 activity (Figure 5B), suggesting that indeed B1R is involved in glutamate-mediated effects on ADAM17 activity and ACE2 shedding.

## 3. Discussion

ACE2 shedding is one of the major contributing mechanisms for the impairment of ACE2 function in cardiovascular, inflammatory, and neurodegenerative diseases. ACE2 shedding is a posttranslational process where the catalytically active ACE2 ectodomain is cleaved from the cell membrane and secreted ACE2 is released into the extracellular environment. Evidence for loss of cellular ACE2 via shedding was observed in various cell lines [10,11,12,26], neurons [8], myocytes [27], pancreatic islets [25], human airway epithelium [28], human cardiac myofibroblasts [29], hypertensive human brains [6], and blood [30] and urine of patients with chronic kidney disease [31]. A prime mediator of ACE2 shedding is ADAM17, also known as tumor necrosis factor-alpha converting enzyme. ADAM17 has been shown to be activated by several mechanisms including pro-inflammatory cytokines, growth factors, oxidative stress, and by G protein-coupled receptors including AT1R. We have previously demonstrated that ACE2 levels within tissues and cells can be modified by post translational mechanisms such as AT1R-dependent internalization processes [9] and AT1R-independent glutamate-induced ACE2 ectodomain shedding via ADAM17 [8]. Recently, we showed direct evidence for involvement of B1R in neuroinflammation and oxidative stress in neurons induced by Ang II stimulation and AT1R activation [32]. However, no prior studies have investigated the role of B1R in altering ADAM17 activity and its effect on ACE2 shedding in neurons. Therefore, in the present study, we investigated the hypothesis that activation of B1R will result in ADAM17-mediated ACE2 shedding in neurons. We demonstrated that both B1R and ADAM17 co-localized in neurons, suggesting the possibility of interaction between B1R and ADAM17. Further, B1R activation increased ADAM17 gene and protein expression, and reduced ACE2 activity in neurons, indicating a role for B1R in ADAM17-mediated ACE2 shedding.

ACE2, ADAM17, and B1R are expressed on neurons within brain nuclei, including the hypothalamus and have been shown to be involved in regulating inflammation and autonomic function during the development of various cardiovascular diseases. ACE2 compensatory activity is reduced in the hypothalamus of hypertensive brains and we previously showed that overexpression of ACE2 specifically within neurons or within the paraventricular nucleus of the hypothalamus attenuates neurogenic hypertension. In addition, we showed that blocking B1R or ADAM17 activation within the brain is beneficial in attenuating hypertension. However, the involvement of B1R activation in ADAM17-mediated ACE2 shedding was not studied previously. A previous study demonstrated that plasma kallikrein can stimulate ADAM17 activity and can lead to transactivation of epidermal growth factor receptor in primary vascular smooth muscle cells, showing evidence for involvement of the kinin system in ADAM17 activation [33]. Indeed, in this study, using a B1R-specific pharmacological antagonist, and gene-deficient neurons, we further confirmed that B1R is responsible for ADAM17-mediated ACE2 shedding.

Glutamate is a major excitatory amino acid involved in enhanced sympathetic drive and inflammation in hypertension. It has been shown that brain hypothalamus contains high levels of glutamate and expresses glutamate receptors [21,34]. Stimulation of glutamatergic neurons in the hypothalamic paraventricular nucleus can cause autonomic dysfunction and elevation of blood pressure suggesting an important role for the glutaminergic system in regulation of neurogenic hypertension [35]. A recent study also demonstrated that activation of ADAM17 in glutamatergic neurons results in the increase in excitability on presympathetic neurons and sympathoexcitation in a salt-sensitive hypertension model [36]. It has been shown that ACE2 does not colocalize with glutamatergic neurons in the paraventricular nucleus, however, glutamatergic excitatory neurons synapse with ACE2-expressing neurons [37]. In contrast, our immunostaining data show that ACE2 is expressed in the primary hypothalamic neurons and co-localizes with the ADAM17 expression. The possible explanation for this observation might be the fact that cultures include neuronal populations from the whole hypothalamus including paraventricular nucleus [37]. Further studies are necessary to characterize the various neuronal populations within the hypothalamus.

Evidence for a possible crosstalk between glutamate-induced excitotoxicity and impairment of ACE2 activity in primary cortical neurons has been reported in our previous study [8]. On the other hand, B1R blockade can reduce neuroinflammation, prevent autonomic dysfunction and sympathoexcitation, and attenuate hypertension in a mouse model of neurogenic hypertension [20]. However, the crosstalk between glutamate and B1R has not been established. Therefore, in the present study, we investigated whether stimulation with glutamate can induce the expression of B1R and facilitate ADAM17-mediated ACE2 shedding in primary hypothalamic neurons. Our data demonstrate that glutamate treatment increased the gene and protein expression of B1R in neurons. Glutamate treatment also increased ADAM17 protein expression and activity, and decreased ACE2 activity in hypothalamic neurons. Furthermore, pretreatment with R715, a B1R-specific antagonist, prevented these glutamate-mediated changes in B1R expression, and ADAM17 and ACE2 activity. These results are further confirmed by treatment of B1RKO neurons with glutamate which did not increase ADAM17 activity or reduce ACE2 activity. Thus, our data clearly suggest that glutamate-mediated effects on ADAM17 activity and ACE2 shedding are mediated by B1R activation in neurons.

**Figure 6:**
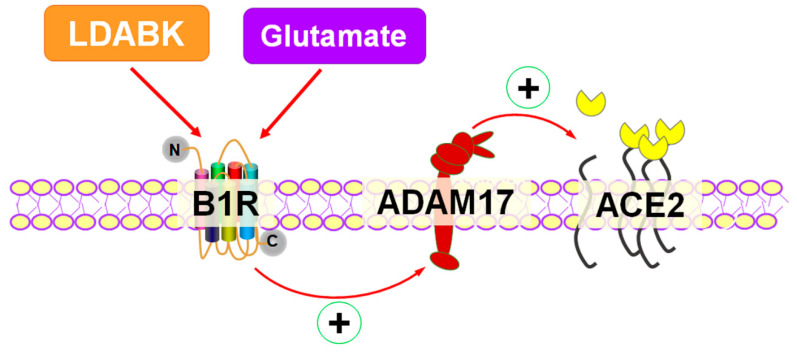
Activation of kinin B1R upregulates ADAM17-mediated ACE2 shedding in primary hypothalamic neurons. B1R activation by specific agonist results in upregulation of ADAM17 expression and activity which results in increased membrane-bound ACE2 shedding and reduced ACE2 activity in primary hypothalamic neurons. In addition, glutamate can induce B1R expression and B1R is involved in glutamate-mediated effects on ADAM17 activity and ACE2 shedding.

In summary, our study demonstrates for the first time that B1R activation results in ADAM17-mediated shedding and reduced ACE2 activity in primary hypothalamic neurons. Moreover, our findings support the novel concept that B1R is involved in glutamate-mediated effects on ADAM17 activity and ACE2 shedding (Figure 6). These findings provide new insights into the role of B1R in decreased compensatory activity of ACE2 in neurons and thus B1R can be a potential therapeutic target in promoting the balance towards beneficiary ACE2/Ang-(1–7) pathway in cardiovascular and neurodegenerative disease conditions.

## 4. Materials and Methods

### 4.1. Primary Neuronal Cell Culture

The experimental protocols used for breeding mice were approved by East Carolina University Animal Care and Use Committee (AUP #W254, approval date: 21 August 2017; #W261, approval date: 16 July 2020) and were performed in accordance with the National Institutes of Health Guidelines for the Care and Use of Laboratory Animals. Kinin B1 receptor knockout (B1RKO) mice were a generous gift from Dr. Michael Bader (Charité Hospital, Berlin, Germany). Wildtype (WT) C57Bl/6NJ mice were purchased from the Jackson Laboratory. Primary hypothalamic neurons were cultured and maintained according to previously published methods [8,32]. Briefly, neonatal or 1-day-old mouse pups were anesthetized with isoflurane (4%) in an oxygen flow (1 L/min) before decapitation and brains were collected in ice-cold Hank’s balanced salt solution (HBSS) (14175-079 Gibco, New York, NY, USA). Hypothalamic tissue was dissected under sterile conditions, rinsed in HBSS, and minced into small pieces using a sterile blade. The minced tissue was transferred into a 15 mL conical tube, washed once with HBSS, then incubated with HBSS containing 1% trypsin (T1426 Sigma-Aldrich, St. Louis, MO, USA) and 1.5 kU/mL DNaseI (D5025 Sigma-Aldrich), digested for 10 min at 37 °C. Then, the tissue was washed with HBSS with 10% FBS twice followed by washing twice with HBSS. The tissue was further triturated in HBSS containing DNase I, using a pipette with 1mL pipette tip (6 times) and then with a 200 μL pipette tip (6 times) attached to a 10 mL serological pipette. Following the disassociation, the cells were spun down by centrifugation and resuspended in complete Neurobasal culture medium supplemented with 2% B27, 0.5 mM GlutaMax and penicillin/streptomycin (100 U/mL and 100 μg/mL, respectively) (Gibco). Dissociated neurons were then plated at a density of 50,000 cells per ml onto poly-L-lysine-coated 6-well plates. The neurons were grown in a humidified atmosphere of 5% CO_2_–95% air at 37 °C. After 24 h, additional fresh medium was added to the cells. On the fourth day, cytosine arabinofuranoside (Ara-C, 2 µM, C1768 Sigma-Aldrich) was added to the neuronal cultures to arrest the growth of non-neuronal cells. Hypothalamic primary neurons were cultured for at least 10 days and then used for further experiments. The treatment durations and doses of LDABK (300 nM), R715 (10 μM), HOE-140 (10 μM), and glutamate (10 μM) are based on our preliminary studies and published literature [8,32].

### 4.2. Immunofluorescence Staining

Primary neurons were grown on poly-L-lysine-coated sterile 15 mm round optically clear borosilicate glass cover slips (#229172, Celltreat, Pepperell, MA, USA) in 12-well plates and fixed with 4% paraformaldehyde for 15 min. The cells were washed with 100 mM Glycine in 1× PBS for 5 min each for 3 times followed by incubating with 0.1% Triton X-100 in 1× PBS for 15 min to permeabilize cells. After blocking with 5% donkey serum in 1× PBS containing 0.1% Tween-20 for 1 h, cells were incubated with MAP2 antibody (#13-1500, lot T5275359, ThermoFisher, Waltham, MA, USA, 1:200 dilution) overnight at 4 °C. Cells were washed with 0.1% Triton X-100 in 1× PBS for 10 min each for 3 times and then incubated with specific secondary antibody (Donkey anti-Mouse Alexa Fluor Plus 488, A32766 Invitrogen, Carlsbad, CA, USA, 1:1000 dilution) for 1 h at room temperature in the dark. Triple immunostaining was performed with specific validated antibodies for detection of ADAM17 (#ab13535, lot GR56873-25, Abcam, Cambridge, UK, 1:500 dilution) and B1R (#ABR-011, lot An-01, Alomone labs, Jerusalem, Israel, 1:200 dilution) or ACE2 ((#OASG00144, lot 1442701, Aviva systems biology, San Diego, CA, USA, 1:200 dilution) coupled with DAPI staining. After 3 washes, coverslips were counterstained with DAPI, then mounted with ProLong Diamond (ThermoFisher) antifade medium. The images were captured with a Zeiss Celldiscoverer 7-cell imaging system.

### 4.3. ACE2 Activity Assay

ACE2 activity was measured from neuronal protein extracts using the fluorogenic substrate Mca-APK(Dnp) as previously described [38,39]. Briefly, the neuronal cells were sonicated in 0.5% Triton X-100 in ACE2 reaction buffer containing 1 M NaCl, 0.5 mM ZnCl_2_, 75 mM Tris HCl and 100 μM Mca-YVADAPK(Dnp). Fluorescence emission at 405 nm, after excitation at 320 nm, was measured and the slope of fluorescence development between 10 and 120 min of incubation was calculated. ACE2 enzyme activities are presented in arbitrary fluorescence units (FU) as amounts of fluorescence substrate converted to florescent product per minute and normalized for total protein.

### 4.4. ADAM17 Activity Assay

ADAM17 activity was measured from neuronal protein extracts using the fluorogenic substrate Dabcyl-SPLAQAVRSSK(5FAM)-NH2 (BioZyme Inc.) as reported previously [8,25]. Briefly, the neuronal protein lysates were prepared in extraction buffer (1 M NaCl, 0.5 mM ZnCl2, and 75 mM Tris HCl, 0.5% Triton X-100, pH 6.5). Equal amounts of protein extracts in 10 μL were incubated in 90 μL of reaction buffer (25 mM Tris-HCl, pH 8.0; 10 mM CaCl_2_) with a final concentration of 5 μM of fluorogenic substrate, with or without addition of 100 nM of the ADAM17-specific inhibitor DPC333. Fluorescence at 530 nm after excitation at 485 nm was measured for 1 h at 37 °C, and slopes in fluorescence development were calculated between the 10- and 60-min time points. Data are presented as fluorescence units (amount of substrate converted to fluorescent product) per minute and normalized for protein content (FU/min/μg).

### 4.5. Gene Expression Analysis by Real Time qRT-PCR

Gene expression was measured using real time RT-PCR as described previously [32]. Total RNA from the primary hypothalamic neurons was extracted using the Direct-Zol RNA miniprep plus kit (Zymo Research, Irvine, CA, USA) according to manufacturer’s protocol. RNA concentration was measured using the spectrophotometer (NanoDrop One; ThermoFisher, Waltham, MA, USA). Real Time PCR amplification reactions were performed with Power SYBR Green RNA-to-CT one-step Kit (Applied Biosystems, Foster City, CA, USA) using a QuantStudio 6 Flex real time PCR machine (Applied Biosystems). PrimeTime qPCR Assay primers (Integrated DNA Technologies, Coralville, IA, USA) for mouse B1R (Primer 1: TCCTGTCCTTCTTCCTTTTGC, Primer 2: AGATCAGAAGCTGCCAAGTTAG, Mm.PT.58.7873555.g, Gene:Bdkrb1, RefSeq: NM_007539) and β-actin (Primer 1: GATTACTGCTCTGGCTCCTAG, Primer 2: GACTCATCGTACTCCTGCTTG, Mm.PT.39a.22214843.g, Gene: Actb, RefSeq: NM_007393) were used. Data were normalized to β-actin expression by the 2^−(∆∆CT)^ comparative method and expressed as a fold change compared to control.

### 4.6. Protein Analysis by Western Blot

Western blots were performed on primary hypothalamic neuronal homogenates, as described previously [20,40]. Neuron samples were homogenized in 1X lysis buffer containing protease and phosphatase inhibitors cocktail (Roche) and incubated on ice for 15 min. Lysates were cleared by centrifugation at 12,000× *g* and 4 °C for 15 min. After determining protein concentration using BCA protein assay kit (Thermo Fisher/Pierce), 15 µg of protein lysates were mixed with Laemmli buffer, heated at 95 °C for 5 min, cooled on ice for 3 min. The samples are resolved on 4–15% Mini-PROTEAN TGX gels (Bio-Rad) under reducing conditions and blotted on to PVDF membranes using Trans-Blot Turbo Transfer system (Bio-Rad). Membranes were blocked with Intercept-TBS blocking buffer (Licor) and immunoblotted overnight at 4 °C with validated antibody against B1R (#ABR-011, Alomone labs). After washing, the membranes were incubated with IRDye secondary antibodies and imaged using Odyssey CLx imaging system (Licor). The density of protein bands was quantitatively analyzed by Image J software (NIH) and expressed as a relative ratio against the loading control.

### 4.7. Statistical Analysis

Statistical analyses were performed using GraphPad Prism 7 (GraphPad Software, San Diego, CA, USA). Data are presented as mean ± standard error of the mean (SEM). Multiple comparisons were made using 1-way analysis of variance followed by Tukey’s multiple comparisons test. Differences were considered statistically significant at *p* < 0.05.

## Data Availability

Data available within the article.

## Abbreviations

ACE2	Angiotensin converting enzyme 2
ADAM17	A Disintegrin And Metalloprotease 17
Ara-C	Cytosine Arabinofuranoside
Ang II	Angiotensin II
AT1R	Angiotensin II type I receptor
B1R	Kinin B1 receptor
LDABK	Lys-Des-Arg^9^-Bradykinin

## References

[1] Xu P., Sriramula S., Lazartigues E. (2011). ACE2/ANG-(1-7)/Mas pathway in the brain: The axis of good. Am. J. Physiol. Regul. Integr. Comp. Physiol..

[2] Jia H., Yue X., Lazartigues E. (2020). ACE2 mouse models: A toolbox for cardiovascular and pulmonary research. Nat. Commun..

[3] Sriramula S., Xia H., Xu P., Lazartigues E. (2015). Brain-targeted angiotensin-converting enzyme 2 overexpression attenuates neurogenic hypertension by inhibiting cyclooxygenase-mediated inflammation. Hypertension.

[4] Sriramula S., Cardinale J.P., Lazartigues E., Francis J. (2011). ACE2 overexpression in the paraventricular nucleus attenuates angiotensin II-induced hypertension. Cardiovasc. Res..

[5] Xia H., Sriramula S., Chhabra K.H., Lazartigues E. (2013). Brain angiotensin-converting enzyme type 2 shedding contributes to the development of neurogenic hypertension. Circ. Res..

[6] Xu J., Sriramula S., Xia H., Moreno-Walton L., Culicchia F., Domenig O., Poglitsch M., Lazartigues E. (2017). Clinical Relevance and Role of Neuronal AT1 Receptors in ADAM17-Mediated ACE2 Shedding in Neurogenic Hypertension. Circ. Res..

[7] Donoghue M., Hsieh F., Baronas E., Godbout K., Gosselin M., Stagliano N., Donovan M., Woolf B., Robison K., Jeyaseelan R. (2000). A novel angiotensin-converting enzyme-related carboxypeptidase (ACE2) converts angiotensin I to angiotensin 1–9. Circ. Res..

[8] Xu J., Sriramula S., Lazartigues E. (2018). Excessive Glutamate Stimulation Impairs ACE2 Activity Through ADAM17-Mediated Shedding in Cultured Cortical Neurons. Cell. Mol. Neurobiol..

[9] Deshotels M.R., Xia H., Sriramula S., Lazartigues E., Filipeanu C.M. (2014). Angiotensin II mediates angiotensin converting enzyme type 2 internalization and degradation through an angiotensin II type I receptor-dependent mechanism. Hypertension.

[10] Lambert D.W., Clarke N.E., Hooper N.M., Turner A.J. (2008). Calmodulin interacts with angiotensin-converting enzyme-2 (ACE2) and inhibits shedding of its ectodomain. FEBS Lett..

[11] Lambert D.W., Yarski M., Warner F.J., Thornhill P., Parkin E.T., Smith A.I., Hooper N.M., Turner A.J. (2005). Tumor necrosis factor-alpha convertase (ADAM17) mediates regulated ectodomain shedding of the severe-acute respiratory syndrome-coronavirus (SARS-CoV) receptor, angiotensin-converting enzyme-2 (ACE2). J. Biol. Chem..

[12] Lai Z.W., Hanchapola I., Steer D.L., Smith A.I. (2011). Angiotensin-converting enzyme 2 ectodomain shedding cleavage-site identification: Determinants and constraints. Biochemistry.

[13] Kuhn J.H., Li W., Choe H., Farzan M. (2004). Angiotensin-converting enzyme 2: A functional receptor for SARS coronavirus. Cell. Mol. Life Sci..

[14] Hofmann H., Pohlmann S. (2004). Cellular entry of the SARS coronavirus. Trends Microbiol..

[15] Zipeto D., Palmeira J.D.F., Arganaraz G.A., Arganaraz E.R. (2020). ACE2/ADAM17/TMPRSS2 Interplay May Be the Main Risk Factor for COVID-19. Front. Immunol..

[16] Sriramula S. (2020). Kinin B1 receptor: A target for neuroinflammation in hypertension. Pharmacol. Res..

[17] Leeb-Lundberg L.M., Marceau F., Muller-Esterl W., Pettibone D.J., Zuraw B.L. (2005). International union of pharmacology. XLV. Classification of the kinin receptor family: From molecular mechanisms to pathophysiological consequences. Pharmacol. Rev..

[18] Regoli D., Barabe J. (1980). Pharmacology of bradykinin and related kinins. Pharmacol. Rev..

[19] Raidoo D.M., Bhoola K.D. (1997). Kinin receptors on human neurones. J. Neuroimmunol..

[20] Sriramula S., Lazartigues E. (2017). Kinin B1 Receptor Promotes Neurogenic Hypertension Through Activation of Centrally Mediated Mechanisms. Hypertension.

[21] Meeker R.B., Greenwood R.S., Hayward J.N. (1994). Glutamate receptors in the rat hypothalamus and pituitary. Endocrinology.

[22] Maher P., Van Leyen K., Dey P.N., Honrath B., Dolga A., Methner A. (2018). The role of Ca(2+) in cell death caused by oxidative glutamate toxicity and ferroptosis. Cell Calcium.

[23] Rueda C.B., Llorente-Folch I., Traba J., Amigo I., Gonzalez-Sanchez P., Contreras L., Juaristi I., Martinez-Valero P., Pardo B., Del Arco A. (2016). Glutamate excitotoxicity and Ca2+-regulation of respiration: Role of the Ca2+ activated mitochondrial transporters (CaMCs). Biochim. Biophys. Acta.

[24] Chaparro-Huerta V., Rivera-Cervantes M.C., Flores-Soto M.E., Gomez-Pinedo U., Beas-Zarate C. (2005). Proinflammatory cytokines and apoptosis following glutamate-induced excitotoxicity mediated by p38 MAPK in the hippocampus of neonatal rats. J. Neuroimmunol..

[25] Pedersen K.B., Chodavarapu H., Porretta C., Robinson L.K., Lazartigues E. (2015). Dynamics of ADAM17-Mediated Shedding of ACE2 Applied to Pancreatic Islets of Male db/db Mice. Endocrinology.

[26] Iwata M., Silva Enciso J.E., Greenberg B.H. (2009). Selective and specific regulation of ectodomain shedding of angiotensin-converting enzyme 2 by tumor necrosis factor alpha-converting enzyme. Am. J. Physiol. Cell Physiol..

[27] Patel V.B., Clarke N., Wang Z., Fan D., Parajuli N., Basu R., Putko B., Kassiri Z., Turner A.J., Oudit G.Y. (2014). Angiotensin II induced proteolytic cleavage of myocardial ACE2 is mediated by TACE/ADAM-17: A positive feedback mechanism in the RAS. J. Mol. Cell Cardiol..

[28] Jia H.P., Look D.C., Tan P., Shi L., Hickey M., Gakhar L., Chappell M.C., Wohlford-Lenane C., McCray P.B. (2009). Ectodomain shedding of angiotensin converting enzyme 2 in human airway epithelia. Am. J. Physiol. Lung Cell Mol. Physiol..

[29] Guy J.L., Lambert D.W., Turner A.J., Porter K.E. (2008). Functional angiotensin-converting enzyme 2 is expressed in human cardiac myofibroblasts. Exp. Physiol..

[30] Lew R.A., Warner F.J., Hanchapola I., Yarski M.A., Ramchand J., Burrell L.M., Smith A.I. (2008). Angiotensin-converting enzyme 2 catalytic activity in human plasma is masked by an endogenous inhibitor. Exp. Physiol..

[31] Xiao F., Hiremath S., Knoll G., Zimpelmann J., Srivaratharajah K., Jadhav D., Fergusson D., Kennedy C.R., Burns K.D. (2012). Increased urinary angiotensin-converting enzyme 2 in renal transplant patients with diabetes. PLoS ONE.

[32] Parekh R.U., Robidoux J., Sriramula S. (2020). Kinin B1 Receptor Blockade Prevents Angiotensin II-induced Neuroinflammation and Oxidative Stress in Primary Hypothalamic Neurons. Cell Mol. Neurobiol..

[33] Abdallah R.T., Keum J.S., El-Shewy H.M., Lee M.H., Wang B., Gooz M., Luttrell D.K., Luttrell L.M., Jaffa A.A. (2010). Plasma kallikrein promotes epidermal growth factor receptor transactivation and signaling in vascular smooth muscle through direct activation of protease-activated receptors. J. Biol. Chem..

[34] Singewald N., Philippu A. (1996). Involvement of biogenic amines and amino acids in the central regulation of cardiovascular homeostasis. Trends Pharmacol. Sci..

[35] Basting T., Xu J., Mukerjee S., Epling J., Fuchs R., Sriramula S., Lazartigues E. (2018). Glutamatergic neurons of the paraventricular nucleus are critical contributors to the development of neurogenic hypertension. J. Physiol..

[36] Xu J., Molinas A.J.R., Mukerjee S., Morgan D.A., Rahmouni K., Zsombok A., Lazartigues E. (2019). Activation of ADAM17 (A Disintegrin and Metalloprotease 17) on Glutamatergic Neurons Selectively Promotes Sympathoexcitation. Hypertension.

[37] Mukerjee S., Gao H., Xu J., Sato R., Zsombok A., Lazartigues E. (2019). ACE2 and ADAM17 Interaction Regulates the Activity of Presympathetic Neurons. Hypertension.

[38] Sriramula S., Pedersen K.B., Xia H., Lazartigues E. (2017). Determining the Enzymatic Activity of Angiotensin-Converting Enzyme 2 (ACE2) in Brain Tissue and Cerebrospinal Fluid Using a Quenched Fluorescent Substrate. Methods Mol. Biol..

[39] Pedersen K.B., Sriramula S., Chhabra K.H., Xia H., Lazartigues E. (2011). Species-specific inhibitor sensitivity of angiotensin-converting enzyme 2 (ACE2) and its implication for ACE2 activity assays. Am. J. Physiol. Regul. Integr. Comp. Physiol..

[40] Ogunlade B., Guidry J.J., Mukerjee S., Sriramula S., Lazartigues E., Filipeanu C.M. (2020). The Actin Bundling Protein Fascin-1 as an ACE2-Accessory Protein. Cell Mol. Neurobiol..

